# Supplementary material for: Host-microbiome protein-protein interactions capture disease-relevant pathways
Source: Genome Biol. 2022 Mar 4;23:72. doi: 10.1186/s13059-022-02643-9 (PMC8895870; doi:10.1186/s13059-022-02643-9)
Supplement: Supplementary file 1 — Additional file 1: Table S1. Extended information on known experimentally verified host-microbiome interactions with evidence for a role in cellular physiology and/or human health. Information on the interaction detection method for human-microbiome PPIs that have been shown to affect cell physiology and/or human health. [file 13059_2022_2643_MOESM1_ESM.pdf]

Table S1. Examples of host-microbiome PPIs that have an experimentally verified role in human cellular physiology and/or disease. Included here are only those proteins with both an experimentally-verified host-microbiome protein-protein interaction and either a physiological effect on host cells or on a disease model that was attributable to the interaction. We excluded proteins found to promote adhesion without direct evidence of specific binding partners or a specific human cellular physiological change; and proteins with evidence of being only in pathogenic strains.

Bacterial protein (UniprotID) (species origin)	Secretion System/ Transmembrane (TM)/ Cell wall (CW) annotation	Human protein	Human protein localization (aggregated for all proteins listed)	Means by which the interaction was verified	Evidence for role in disease	Detection and Disease-association
Amuc_1100 (B2UR41) (*Akkermansia muciniphila*)	TM	Toll-like receptor 2 (TLR2)	Membrane	Biolayer interferometry PMID: 32736072	IL-1β, IL-6, IL-8, IL-10 and TNF-α production in PBMCs; increase in barrier function; PMID: 28249045 improve glucose tolerance and induce a lower body weight and fat mass gain in mice fed a high-fat diet PMID: 27892954	Disease-associated
Enolase (EnoA1) (Q88YH3) (*Lactobacillus plantarum* LM3)	No annotation	Plasminogen	Membrane, Extracellular, Intracellular	Bidimensional electrophoresis, plasminogen overlay assay, pull down using saturation binding PMID: 23103380	Enhancement of tissue-type plasminogen activator (tPA)-mediated conversion of plasminogen to plasmin PMID: 23103380	Disease-associated
FadA (Q5I6B0) (*Fusobacterium nucleatum*)	Sec	E-cadherin (Cadherin-1)	Membrane, Extracellular, Intracellular	Western blot and synthetic peptide based on E-cadherin binding domain that abrogates FadA binding. PMID: 23954158	Stimulates proliferation of human CRC cell lines (verified using complementation tests). Activates β-catenin signaling pathways. PMID: 23954158	Not disease-associated using this method
Faf (Q2TV77) (*Finegoldia magna*)	Sec, TM, CW	Histones H4 and H2B	Membrane, Extracellular, Intracellular	Western blot, IP-MS PMID: 24335013	Binds histones and prevents antibacteriocidal activity.	Not detected

					PMID: 24335013	
Fap2<br>(A0A0X3Y2Y3)<br>(*Fusobacterium nucleatum*)	Sec, TM	T cell immunoreceptor with Ig and ITIM domains (TIG IT)	Membrane	ELISA-based binding assays and Western blot analysis. Verified with truncation mutations.<br>PMID: 25680274	Inhibits natural killer (NK) and tumor infiltrating lymphocyte (TIL) cytotoxicity and hemagglutination of red blood cells. Verified by transposon mutants.<br>PMID: 25680274	Not detected
FimH<br>(P08191)<br>(commensal *Escherichia coli*)	Sec, TM	Pancreatic secretory granule membrane major glycoprotei n GP2	Membrane, Extracellular, Intracellular	Binding confirmed with knockout<br>PMID: 19907495<br>Affinity mass spectrometry using derivatives of commensal K-12<br>PMID: 19627615	Initiates mucosal immune response via M cells<br>PMID: 19907495	Not disease-associated using this method
Flagellin (FliC)<br>(P06179)<br>*Salmonella enterica*<br>Note: Direct binding has only been demonstrated for *Salmonella typhimurium*, though flagellin from commensal Firmicutes stimulates TLR5.<br>PMID: 24237702	T3SS	Toll-like receptor 5 (TLR5)<br>PMID:14625549	Membrane, Intracellular	Co-immunoprecipitation, alanine scanning<br>PMID:14625549	Induces MyD88-dependent signaling and activation of NF- κB.<br>PMID: 11323673<br>Gut microbiome involvement shown in TLR-/- mice<br>PMID: 24237702	Not detected
GelE<br>(Q833V7)<br>(*Enterococcus faecalis)*	Sec, TM	Glucagon like peptide 1 (GLP-1), gastric inhibitory polypetide, glucagon, leptin, PPY, PYY. MCP-1, TNF-α<br>PMID: 32051237; mouse E-cadherin<br>PMID: 21699778; C3 and iC3b<br>PMID: 18941224	Membrane, Extracellular, Intracellular	Specificity for cleavage of GLP-1, confirmed by knock-out<br>PMID: 32051237	Barrier function measured by TEER (absent in knockout)<br>PMID: 32051237<br>Contributes to intestinal inflammation (confirmed with inhibitors)<br>PMID: 21699778	Not disease-associated using this method

Microbial anti-inflammatory molecule (MAM) (C7H4X2) (*Faecalibacterium prausnitzii*)	No annotation	ZO-1, DDX3X, ANXA2, FASN, FLNA, FLOT2, HSP90AB1, HSPA1B, JUP, KRT18, MYH9, PRDX1, PUF60, RACK1, RSL1D1, RPL14, RPL24, YWHAZ	Membrane, Extracellular, Intracellular	Affinity pull-down PMID: 31503404	Improves barrier function *in vitro* and *in vivo*; increases ZO-1 transcription; PMID: 31503404 Inhibits NF-KB signaling; PMID: 26045134	12/18 human interactors are disease-associated.
Mub (A0A0S2Z342) (*Lactobacillus plantarum*)	T3SS	Cytokeratins (1, 4, 5, 6, 8, 9, 10), Hsp90, Laminin	Membrane, Extracellular, Intracellular	Affinity purification followed by mass spectrometry PMID: 30242281	Administration of the binding portion (Mubs5s6) results in pathogenic exclusion (decrease in the adhesion of enterotoxigenic *E. coli*) to intestinal epithelial cells. PMID: 30242281	Not detected.
p9 (Amuc_1631) (B2UM07) (*Akkermansia muciniphila*)	T3SS, Sec	Intercellular adhesion molecule 2 (ICAM2)	Membrane	Ligand-receptor capture analysis with LC-MS PMID: 33820962	Protein increases glucagon-like peptide-1 secretion and brown adipose tissue thermogenesis. Verified by IL-6 deficiency, which downregulates ICAM-2 expression. PMID: 33820962	Disease-associated
SlpA (P35829) (*Lactobacillus acidophilus* NCFM)	T4SS, Sec, TM, CW	CD209 antigen (DC-specific ICAM-3-grabbing nonintegrin (DC-SIGN))	Membrane, Extracellular, Intracellular	ELISA identified binding partners; confirmed with *SplA* knockout and with anti-DC-SIGN antibodies. PMID:19047644	Th2 polarization of dendritic cells and induction of IL-4 expression PMID: 19047644	Not detected
