# Supplementary material for: Host-microbiome protein-protein interactions capture disease-relevant pathways
Source: Genome Biol. 2022 Mar 4;23:72. doi: 10.1186/s13059-022-02643-9 (PMC8895870; doi:10.1186/s13059-022-02643-9)
Supplement: Supplementary file 2 — Additional file 2.. This file contains Figures S1-S14. [file 13059_2022_2643_MOESM2_ESM.pdf]

Supplemental Note

Terminology used for gene-disease associations

The following terms from DisGeNet were used for each of the following broader disease annotations. For diabetes, we included all subtypes and diabetes-related phenotypes.

CRC: 'Colorectal Carcinoma', 'Colorectal Neoplasms', 'Adenocarcinoma of large intestine', 'Malignant tumor of colon', 'Hereditary Nonpolyposis Colorectal Neoplasms', 'Hereditary non-polyposis colorectal cancer syndrome', 'Hereditary Nonpolyposis Colorectal Cancer', 'Colorectal cancer, hereditary nonpolyposis, type 1', 'Hereditary nonpolyposis colorectal carcinoma', 'Colon Carcinoma', 'Colorectal Cancer, Susceptibility to, 4', 'Colorectal Cancer, Susceptibility to, on Chromosome 15', 'Colorectal Cancer, Hereditary Nonpolyposis, type 7 (disorder)', 'Colorectal Cancer, Hereditary Nonpolyposis, type 5', 'Colorectal Cancer, Hereditary Nonpolyposis, type 8', 'Colorectal Adenomatous Polyposis, Autosomal Recessive', 'Colorectal Cancer, Hereditary Nonpolyposis, type 4', 'Colorectal Cancer, Susceptibility to, 10', 'Colorectal Cancer, Susceptibility to, 12', 'Familial Colorectal Cancer Type X', 'Colorectal Cancer, Hereditary Nonpolyposis, type 6', 'Colorectal Cancer, Susceptibility to, 1', 'Oligodontia-Colorectal Cancer Syndrome'

Diabetes: 'Diabetes Mellitus, Experimental', 'Diabetic Nephropathy', 'Diabetes Mellitus, Non-Insulin-Dependent', 'Diabetes Mellitus, Insulin-Dependent', 'Diabetes, Autoimmune', 'Brittle diabetes', 'Diabetes Mellitus, Ketosis-Prone', 'Diabetes Mellitus, Sudden-Onset', 'Diabetic Retinopathy', 'Diabetic Cardiomyopathies', 'Diabetic cystopathy', 'Diabetes Mellitus', 'Complications of Diabetes Mellitus', 'Neonatal diabetes mellitus', 'Gestational Diabetes', 'Alloxan Diabetes', 'Streptozotocin Diabetes', 'Prediabetes syndrome', 'Diabetic Angiopathies', 'Microangiopathy, Diabetic', 'Diabetes Mellitus, Noninsulin-dependent, 1 (disorder)', 'Diabetic Neuropathies', 'Symmetric Diabetic Proximal Motor Neuropathy', 'Asymmetric Diabetic Proximal Motor Neuropathy', 'Diabetic Mononeuropathy', 'Diabetic Polyneuropathies', 'Diabetic Amyotrophy', 'Diabetic Autonomic Neuropathy', 'Diabetic Asymmetric Polyneuropathy', 'Diabetic Neuralgia', 'Nephrogenic Diabetes Insipidus', 'Diabetes Mellitus, Insulin-Dependent, 22 (disorder)', 'Microcephaly, Epilepsy, and Diabetes Syndrome', 'Diabetes', 'Diabetes Mellitus, Insulin-Dependent, 12', 'Microvascular Complications of Diabetes, Susceptibility to, 3 (finding)', 'Diabetes Mellitus, Neonatal, with Congenital Hypothyroidism', 'Phosphate Diabetes', 'Diabetic encephalopathy', 'Microvascular Complications of Diabetes, Susceptibility to, 2 (finding)', 'Insulin-resistant diabetes mellitus', 'Lymphedema-Distichiasis Syndrome with Renal Disease and Diabetes Mellitus', 'Lipoatrophic Diabetes Mellitus', 'Pregnancy in Diabetics', 'Maturity onset diabetes mellitus in young', 'Maturity-Onset Diabetes of the Young, type 14', 'Latent Autoimmune Diabetes in Adults', 'Monogenic diabetes', 'Diabetes mellitus autosomal dominant type II (disorder)', ' Diabetes Mellitus, Permanent Neonatal', 'Diabetes Insipidus', Microvascular Complications of OF Diabetes, Susceptibility to, 7 (finding)', 'Renal cysts and diabetes syndrome', 'Maturity-Onset Diabetes of the Young, Type 1', 'Fanconi Renotubular Syndrome 4 with Maturity-onset Diabetes of the Young', 'Transient neonatal diabetes mellitus', 'Diabetes Mellitus, Transient Neonatal, 1', 'Diabetes Mellitus, Insulin-Dependent, 2', 'diabetes (mellitus) due to autoimmune process', 'Diabetes (mellitus) due to immune mediated pancreatic islet beta-cell destruction', 'Idiopathic Diabetes (Mellitus)', Microvascular Complications of Diabetes, Susceptibility to, 4 (finding)', 'Diabetes Mellitus, Insulin-Dependent, 10', 'Acquired Nephrogenic Diabetes Insipidus', 'Congenital Nephrogenic Diabetes Insipidus', 'Nephrogenic Diabetes Insipidus, Type I', 'Nephrogenic Diabetes Insipidus, Type II', 'ADH-Resistant Diabetes Insipidus', 'Diabetic Ketoacidosis', 'Non-insulin-dependent diabetes mellitus with unspecified complications', 'Diabetes Mellitus, Permanent Neonatal, with Neurologic Features', 'Developmental Delay, Epilepsy, and Neonatal Diabetes', 'Maturity-onset diabetes of the young, type 10', 'Diabetes Mellitus, Insulin-Resistant, with Acanthosis Nigricans', 'Maturity-onset Diabetes of the Young, type IV (disorder)', 'Diabetes Mellitus, Transient Neonatal, 3 (disorder)', 'Maturity-onset Diabetes of the Young, type 13', 'Diabetes Mellitus, Insulin-Dependent, 5', ' Diabetes Mellitus, Insulin-Dependent, 7',

‘Maturity-onset Diabetes of the Young, type 6 (disorder)’, ‘Gastroparesis with diabetes mellitus’, ‘Other specified diabetes mellitus with unspecified complications’, ‘Insulin-dependent diabetes mellitus secretory diarrhea syndrome’, ‘Severe nonproliferative diabetic retinopathy’, ‘Microvascular Complications of Diabetes, Susceptibility to, 5 (finding)’, ‘Central Diabetes Insipidus’, ‘Ataxia, Combined Cerebellar and Peripheral, with Hearing Loss and Diabetes Mellitus’, ‘Maturity-onset diabetes of the young, type 11’, ‘Microvascular Complications of Diabetes, Susceptibility to, 6 (finding)’, ‘Diabetes Mellitus, Transient Neonatal, 2 (disorder)’, ‘Maturity-onset Diabetes of the Young, type 3 (disorder)’, ‘Diabetes Mellitus, Insulin-Dependent, 20 (disorder)’, ‘Proliferative diabetic retinopathy’, ‘Microvascular Complications of Diabetes, Susceptibiltiy to, 1(finding)’, ‘Maturity-onset Diabetes of the Young, type type 7 (disorder)’, ‘Diabetes Mellitus, Noninsulin-dependent, 5’

Autoimmune: ‘Autoimmune hemolytic anemia’, ‘Autoimmune Diseases’, ‘Autoimmune state’, ‘Celiac Disease’, ‘Lupus Erythematosus, Systemic’, ‘Diabetes, Autoimmune’, ‘Autoimmune Chronic Hepatitis’, ‘Rheumatoid Arthritis’, ‘Ankylosing spondylitis’, ‘Multiple Sclerosis’, ‘Autoimmune Lymphoproliferative Syndrome’, ‘Experimental Autoimmune Encephalomyelitis’, ‘Lupus Erythematosus, Cutaneous’, ‘Chilblain lupus 1’, ‘Multiple Sclerosis, Acute Fulminating’, ‘Autoimmune thyroiditis’, ‘Autoimmune Lymphoproliferative Syndrome Type 2B’, ‘Autoimmune Interstitial Lung, Joint, and Kidney Disease’, ‘Lupus Vulgaris’, ‘Lupus Erythematosus, Discoid’, ‘Lupus Erythematosus’, ‘Rheumatoid Arthritis, Systemic Juvenile’, ‘Neuritis, Autoimmune, Experimental’, Systemic Lupus Erythematosus 16’, ‘Ankylosing spondylitis and other inflammatory spondylopathies’, ‘Lupus Vasculitis, Central Nervous System’, ‘Lupus Meningoencephalitis’, ‘Neuropsychiatric Systemic Lupus Erythematosus’, ‘Lupus Nephritis’, ‘Vitiligo-associated Multiple Autoimmune Disease Susceptibility 1 (finding)’, Chilblain Lupus 2’, ‘Latent Autoimmune Diabetes in Adults’, ‘Vitiligo-associated Multiple Autoimmune Disease Susceptibility 6’, ‘Autoimmune Disease, Susceptibility to, 1’, ‘Autoimmune Hepatitis with Centrilobular Necrosis’, ‘Polyendocrinopathies, Autoimmune’, ‘Polyglandular Type I Autoimmune Syndrome’, ‘Autoimmune Syndrome Type II, Polyglandular’, ‘Polyglandular Type III Autoimmune Syndrome’, ‘Autoimmune Polyendocrinopathy Syndrome, Type I, Autosomal Dominant’, ‘Autoimmune Polyendocrinopathy Syndrome, type I, with Reversible Metaphyseal Dysplasia’, ‘Autoimmune polyendocrinopathy syndrome, type 1’, ‘Multiple Sclerosis, Acute Relapsing’, ‘Multiple Sclerosis, Relapsing-Remitting’, ‘diabetes (mellitus) due to autoimmune process’, ‘Autoimmune Lymphoproliferative Syndrome, Type IA’, ‘Ras-associatedAutoimmune Leukoproliferative Disorder’, ‘Autoimmune Lymphoproliferative Syndrome Type 1, Autosomal Dominant’, ‘Autoimmune Diseases of the Nervous System’, ‘Autoimmune Disease, Susceptibility to, 6’, ‘Autoimmune Lymphoproliferative Syndrome, Type III’, ‘Alpha/Beta T-cell Lymphopenia with Gama/Delta T-cell Expansion, Severe Cytomegalovirus Infection, and Autoimmunity, ‘Idiopathic Autoimmune Hemolytic Anemia’, ‘Autoimmune Disease, Multisystem, Infantile-onset, 1’, ‘Systemic Lupus Erythematosus, Multisystem, 11’, ‘T-cell Immunodeficiency, Recurrent Infections, and Autoimmunity with or without Cardiac Malformations’, ‘T-cell Immunodeficiency, Recurrent Infections, Autoimmunity, and Cardiac Malformations’, ‘Hyperthyroidism, Nonautoimmune’, ‘Autoimmune Disease, Multisystem, Infantile-onset, 2’, ‘Autoimmune Disease, Multisystem, with facial dysmorphism’, ‘Syndromic multisystem autoimmune disease due to itch deficiency’, ‘Autoimmune Lymphoproliferative Syndrome, Type IIA’, ‘Immunodeficiency, Common Variable, 8 with Autoimmunity’

Obesity: ‘Obesity’, ‘Pediatric Obesity’, ‘Adolescent Obesity’, ‘Childhood Overweight’, ‘Infantile Obesity’, ‘Infant Overweight’, ‘Adolescent Overweight’, ‘Abdominal obesity metabolic syndrome’, ‘Obesity, Morbid’, ‘Obesity, Hyperphagia, and Developmental Delay’, ‘Obesity, Abdominal’, ‘Mental Retardation, Epilectic Seizures, Hypogonadism and Hypogenitalism, Microcephaly, and Obesity (disorder)’, ‘Obesity, Susceptibility to’, ‘Obesity, Visceral’, ‘Overweight’, ‘Obesity due to melanocortin 4 receptor deficiency’, ‘ABDOMINAL Obesity-Metabolic Syndrome 1’, ‘Developmental Delay, Intellectual Disability, Obesity, and Feautres’, ‘Spastic Paraplegia, Intellectual disability, nystagmus, and

Obesity', 'Retinal Dystrophy and Obesity ', 'Childhood-onset truncal obesity', 'Morbid Obesity and Spermatogenic Failure', 'Abdominal Obesity-Metabolic Syndrome 3'

IBD: 'Ulcerative Colitis', 'Crohn Disease', 'Colitis', "Crohn's disease of large bowel", 'Inflammatory Bowel Diseases', 'Necrotizing Enterocolitis', "Crohn's disease of the ileum", 'IIeocolitis', 'Inflammatory Bowel Disease 17', 'Chronic left-sided ulcerative colitis', 'Inflammatory Bowel Disease 12', 'Inflammatory Bowel Disease 19', 'Enterocolitis', 'Enterocolitis, Neutropenic', 'Inflammatory bowel disease 28, Autosomal Recessive', 'Inflammatory bowel disease 25, autosomal recessive', 'Inflammatory Bowel Disease 14', 'Inflammatory Bowel Disease 13', 'Inflammatory Bowel Disease 10','Inflammatory Bowel Disease 29', 'Autoinflammation with Infantile Enterocolitis' 'Crohn Disease-associated Growth Failure, Susceptibility to (finding)', 'Neutropenic colitis', ;Inflammatory Bowel Disease, Immunodeficiency, and encephalopathy', 'Inflammatory Bowel Disease, Immunodeficiency, and Encephalopathy', 'Inflammatory Bowel Disease 16'

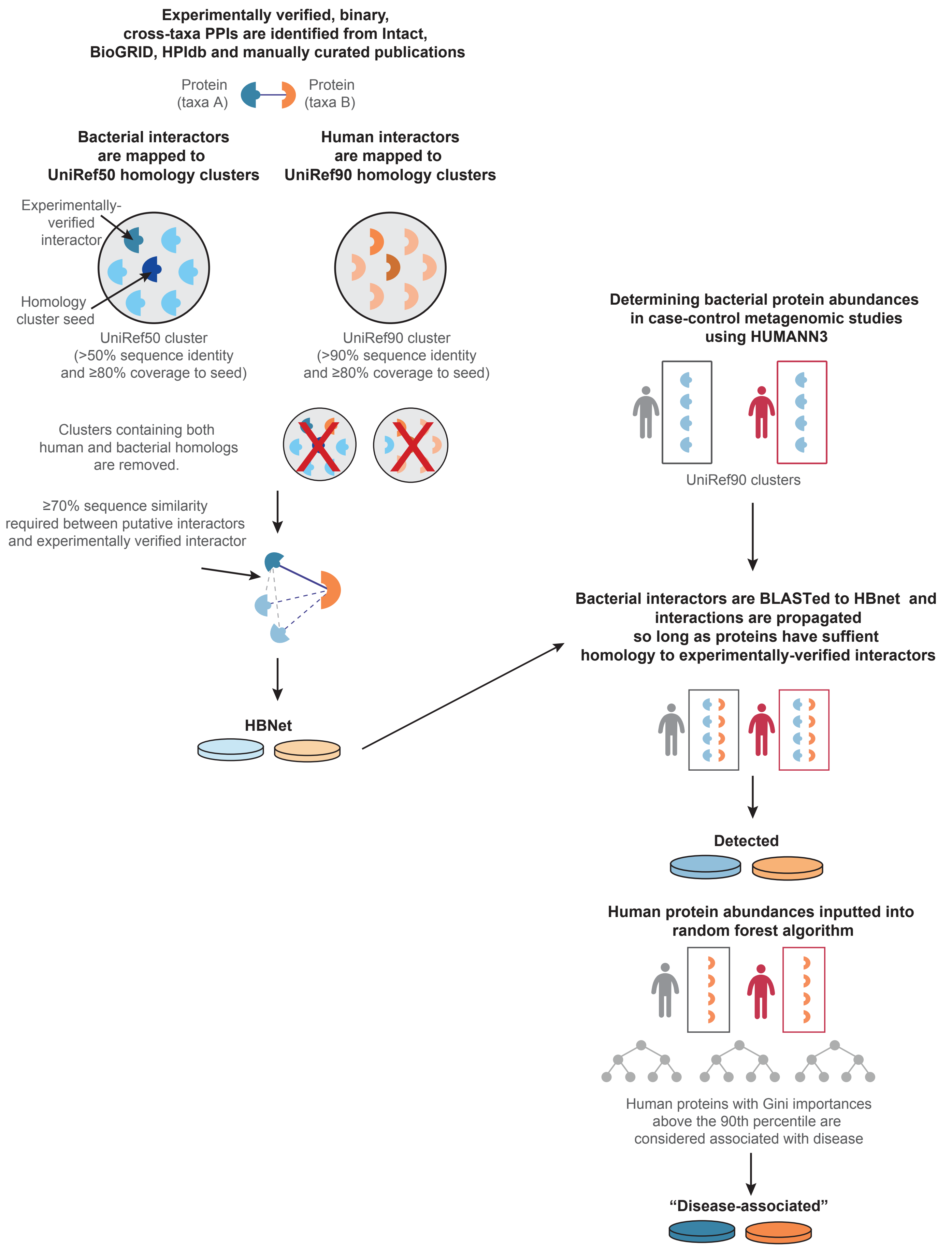

Figure S1. An outline of our homology mapping procedure and alignment.
Depiction of the interaction network inference and protein detection pipeline for bacterial/microbiome (blue)-human (orange) PPIs.

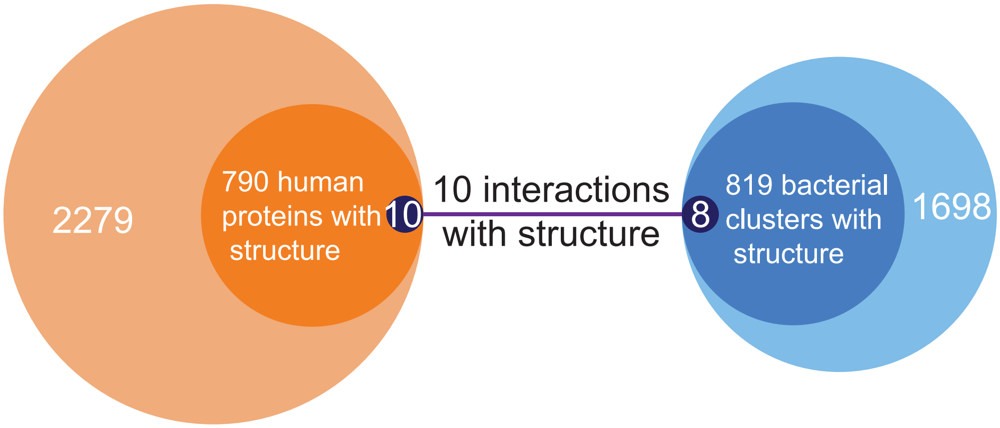

Figure S2. Few bacterial-human interaction sequences populate the Protein Data Bank.
A Venn diagram describing the number of detected bacterial clusters and human interactors in the nine metagenomic cohorts that have any matching structure (using BLASTp) in the PDB to at least one chain (medium blue) and whether their homologous structures appear on the same PDB cocrystal structure (dark blue). Only 10 PDB structures showed non-overlapping homology to both a human and bacterial protein.

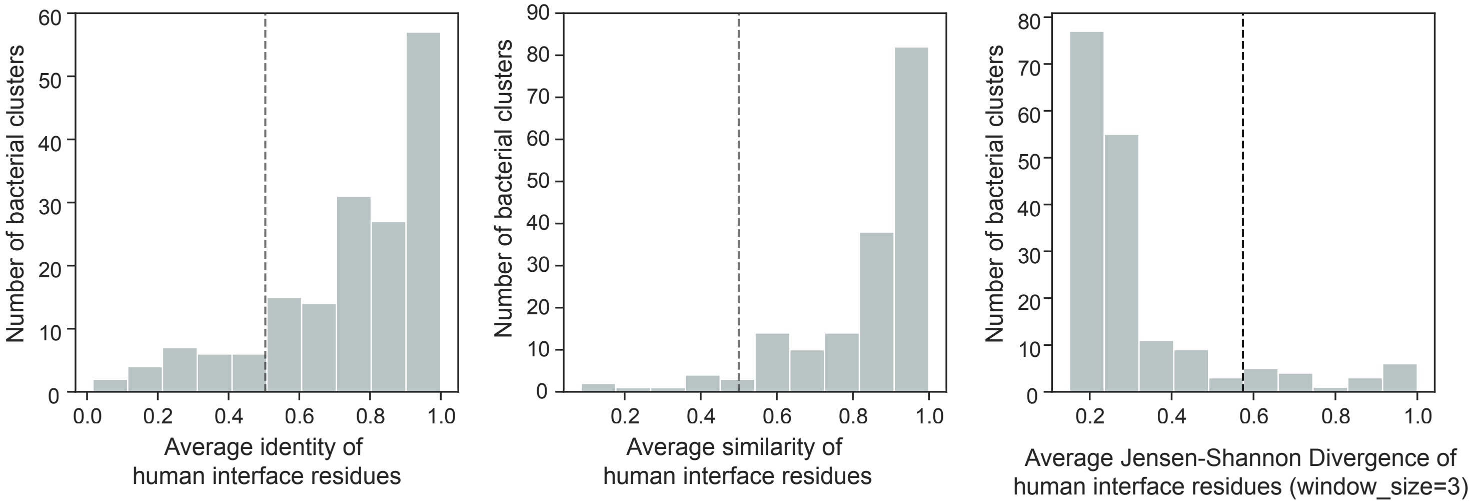

Figure S3. Interface similarity between bacterial proteins within a UniRef cluster. Similarity, identity, and Jensen-Shannon divergence of interface residues across all bacterial members of the same UniRef cluster sourced from all cocrystal structures in the PDB with human and bacterial interactors.

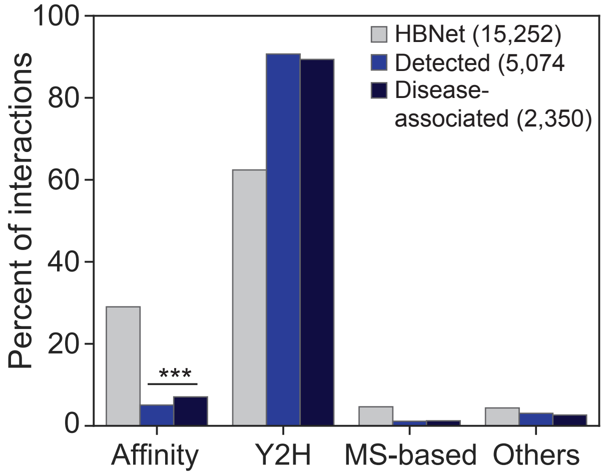

Figure S4. Disease-associated interactions are enriched for those based on affinity-based methods.
The three largest categories of detection methods are shown (affinity-based methods, yeast-2-hybrid, mass spectrometry methods) as well as 'Other'. p-values are only shown between 'Detected' and 'Disease-associated' and are depicted by: * p<0.05; ** p<0.01; *** p<0.001; **** p<0.0001 (Chi-square test). Total numbers of each set are noted in the legend.

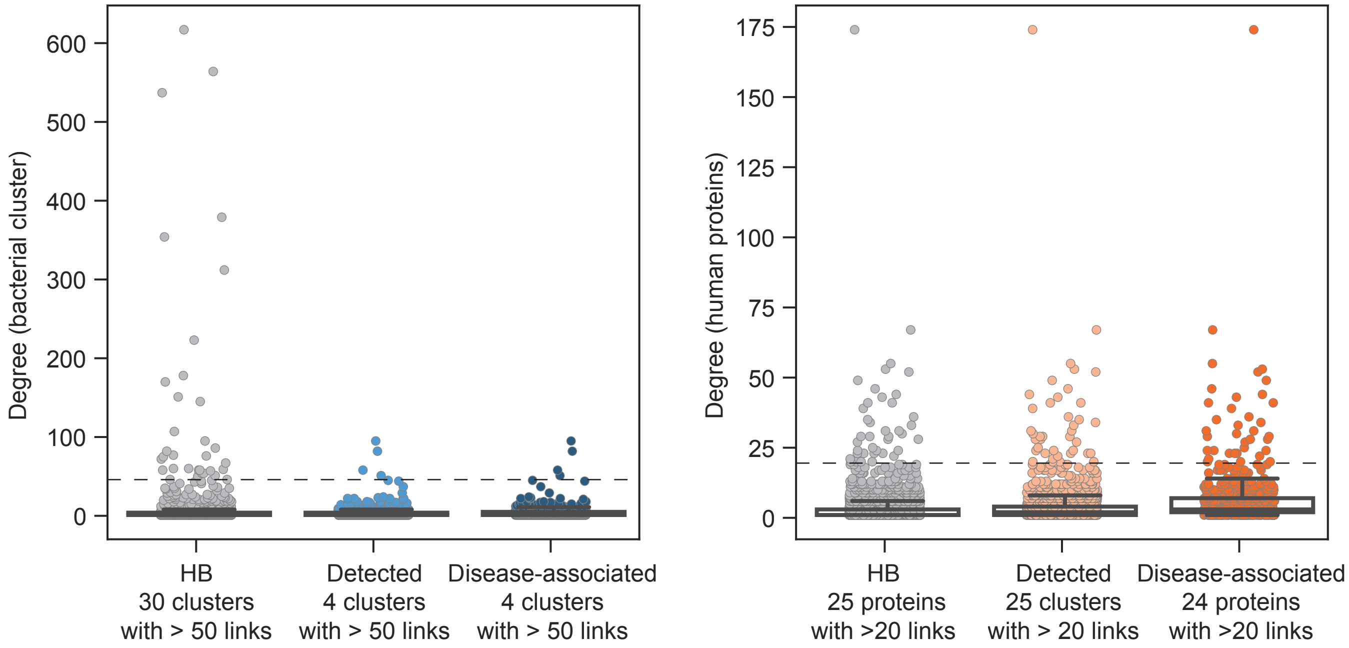

Figure S5. Degree distribution for bacterial protein clusters and human proteins.
The degree distribution per bacterial protein cluster (left) or human protein (right) in the HBNet, Detected or Disease-associated subsets.

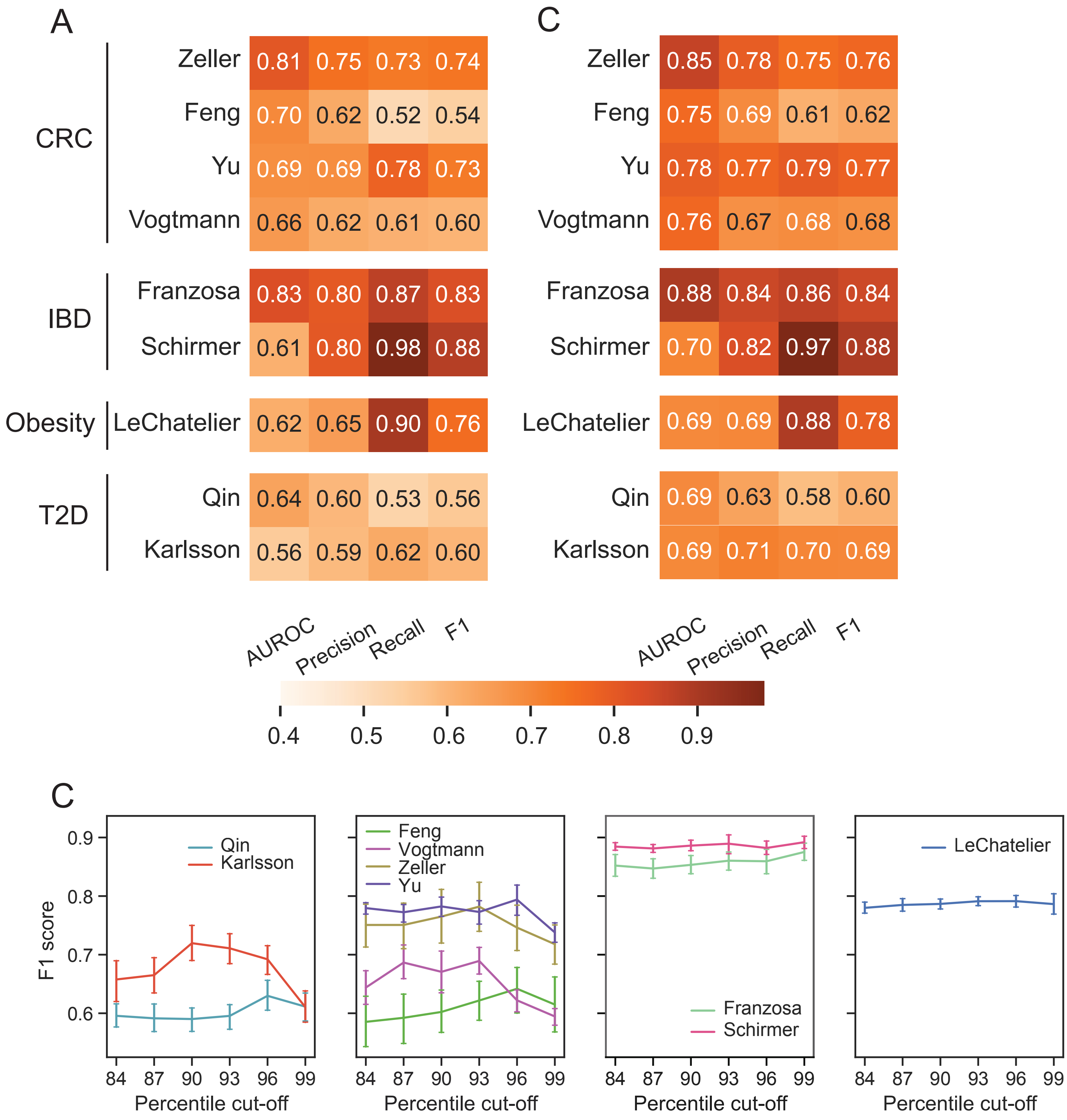

Figure S6. Performance metrics of the random forest (RF) classifier.

(A) A heatmap of area under the receiver operating characteristic curve (AUROC), precision, recall, and F1-scores for random forests on the putative human interactors with the microbiomes of each metagenomic study with grid search-based hyper-parameter tuning, evaluated using five-fold cross validation. **(B)** Performance metrics of the RF classifier using only features above the 90th percentile. **(C)** F1 scores of RF classifiers using features above different percentile cutoffs. Error bars are 70% confidence intervals.

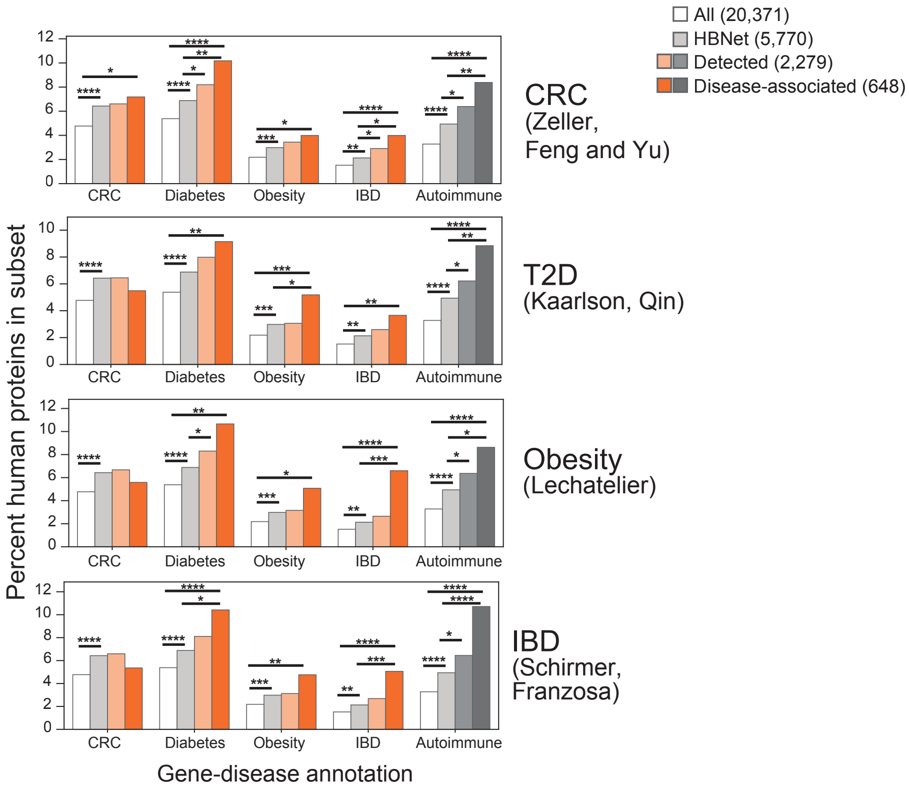

Figure S7. Gene-disease annotations are specific to each disease cohort. The proportions of human proteins implicated in disease, according to their GDAs in DisGeNET (only GDAs with scores over 0.1 were considered) and grouped according to disease-specific cohorts, in the following subsets: all reviewed human proteins (totaling 20,371 proteins); HBNet (5,770 proteins); human interactors with detected bacterial proteins (2,279 proteins); and those human interactors with feature importances above the 90th percentile in their respective cohorts (648 unique proteins). p-values are depicted by: * $p<0.05$; ** $p<0.01$; *** $p<0.001$; **** $p<0.0001$ (Chi-square test). Total numbers of each set are noted in the legend.

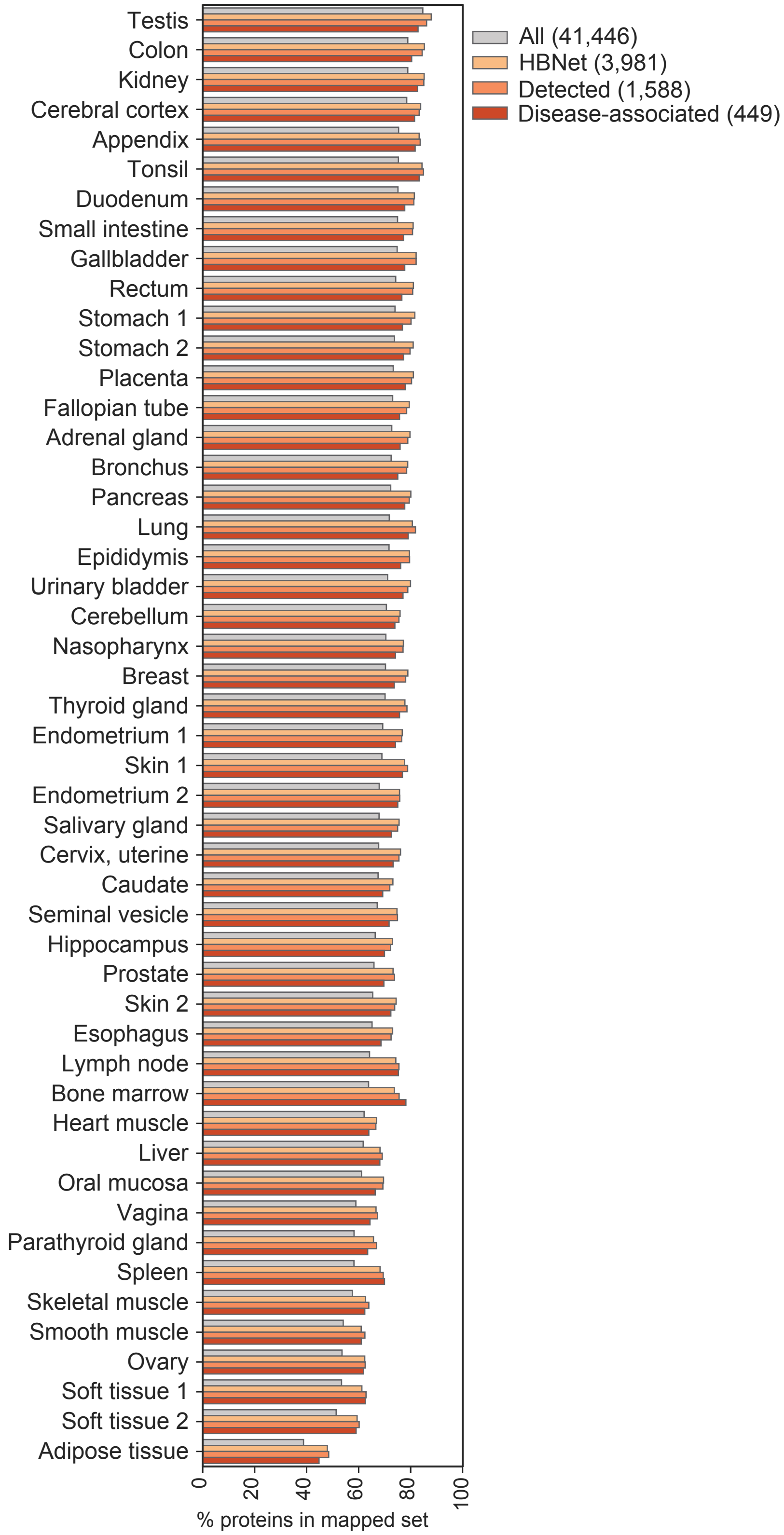

Figure S8. Protein localization and protein expression according to human tissue.
Protein localization according to tissue, as annotated by the Human Protein Atlas. Only those with “enhanced”, “supported” or “approved” annotations were included. Total numbers of each set are noted in the legend.

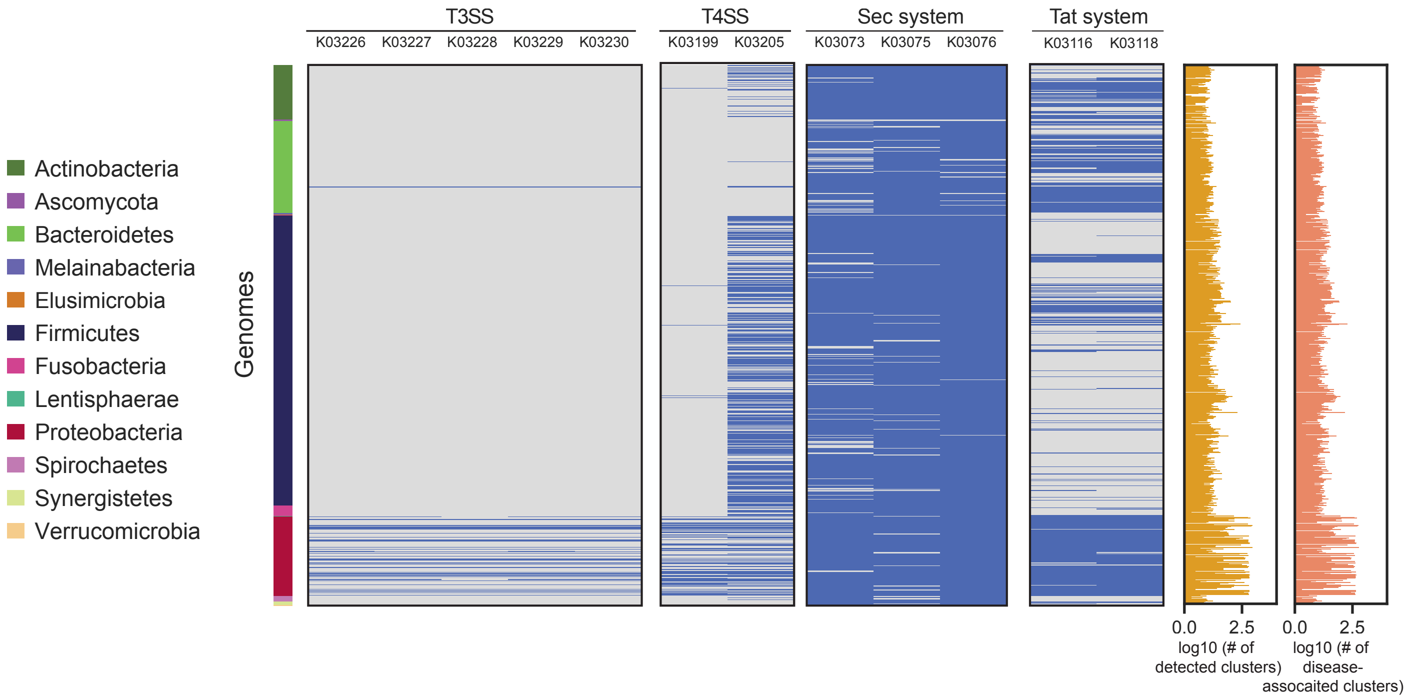

Figure S9. Secretion systems distribution varies across bacterial species.
A heatmap (present/absent) of the required components for each secretion system (denoted using their KO numbers) present in each bacterial species (colored by phylum to the left) with at least one detected protein associated with bacterial protein clusters in nine case-control cohort studies. The actual number of detected and disease-associated protein cluster representatives for each bacteria in any of the nine metagenomic studies is plotted to the right.

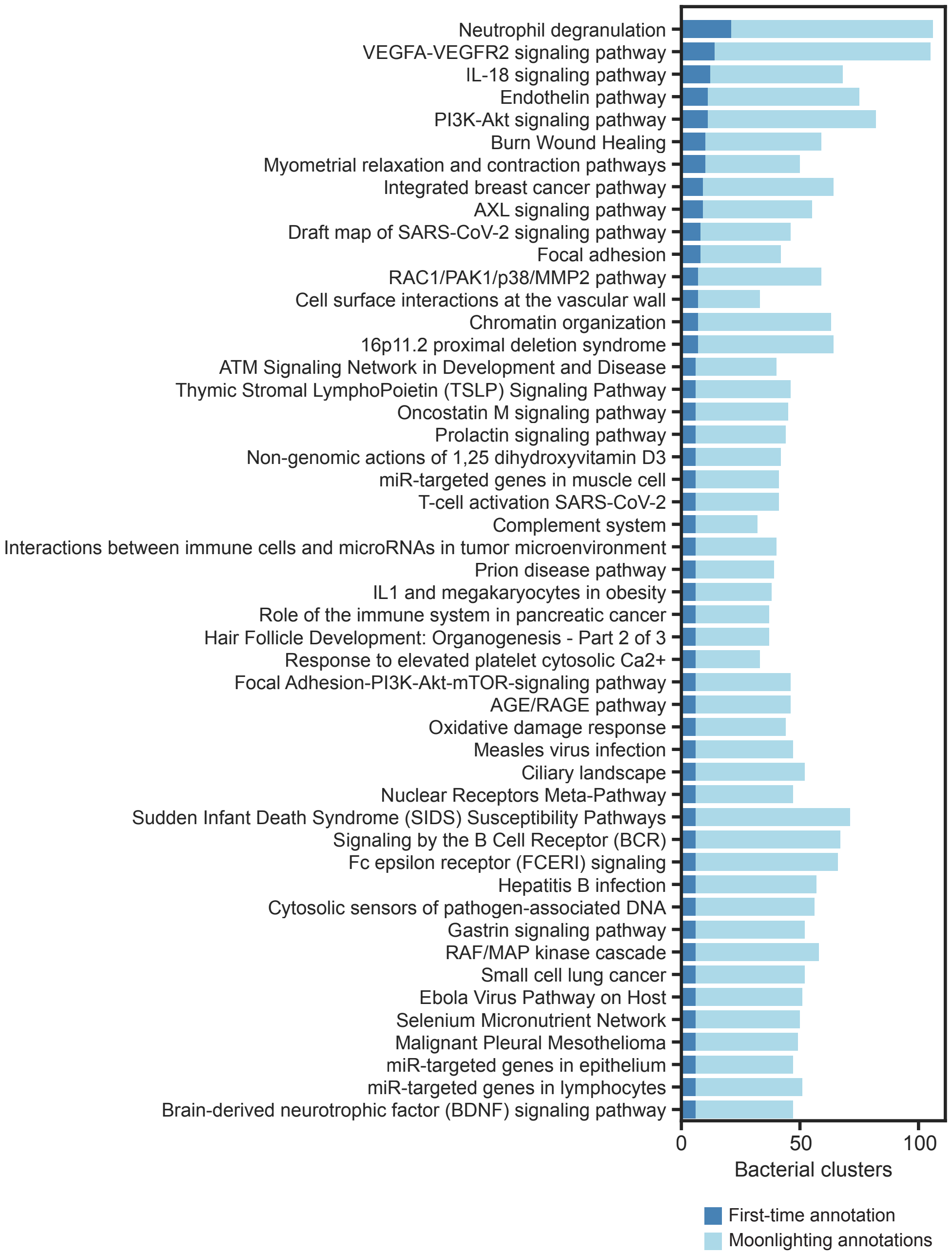

Figure S10. Bacterial clusters gain putative human-relevant functions.
Human pathways (annotated using WikiPathways) significantly enriched (FDR-adjusted p-values < 0.05) in either HBNet, the human proteins targeted by bacterial clusters detected in the metagenomic studies, or those human targets associated with disease in the metagenomic case-control cohort studies (disease-associated). 953 out of 1,102 metagenomic cohort-associated human proteins were able to be annotated. Note that each bacterial protein cluster may gain multiple annotations, according to the roles of their human interactor(s).

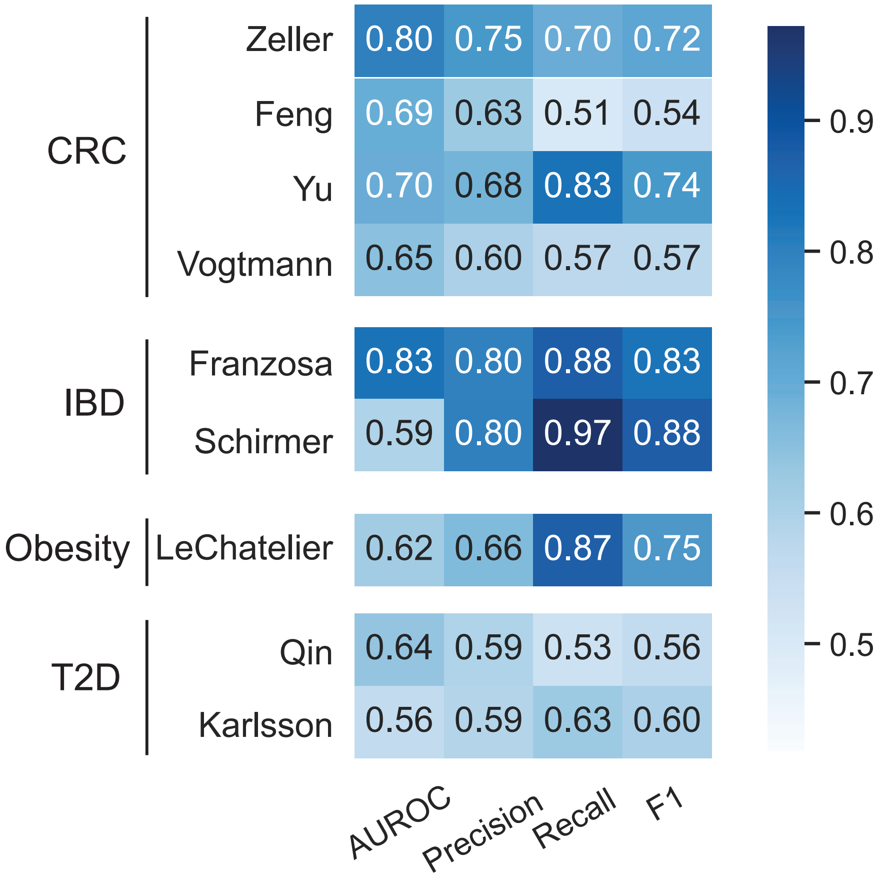

Figure S11. Performance metrics of the random forest (RF) classifier using bacterial abundances. A heatmap of area under the receiver operating characteristic curve (AUROC), precision, recall, and F1-scores for random forests on the putative bacterial interactors of each metagenomic study with grid search-based hyper-parameter tuning, evaluated using five-fold cross validation.

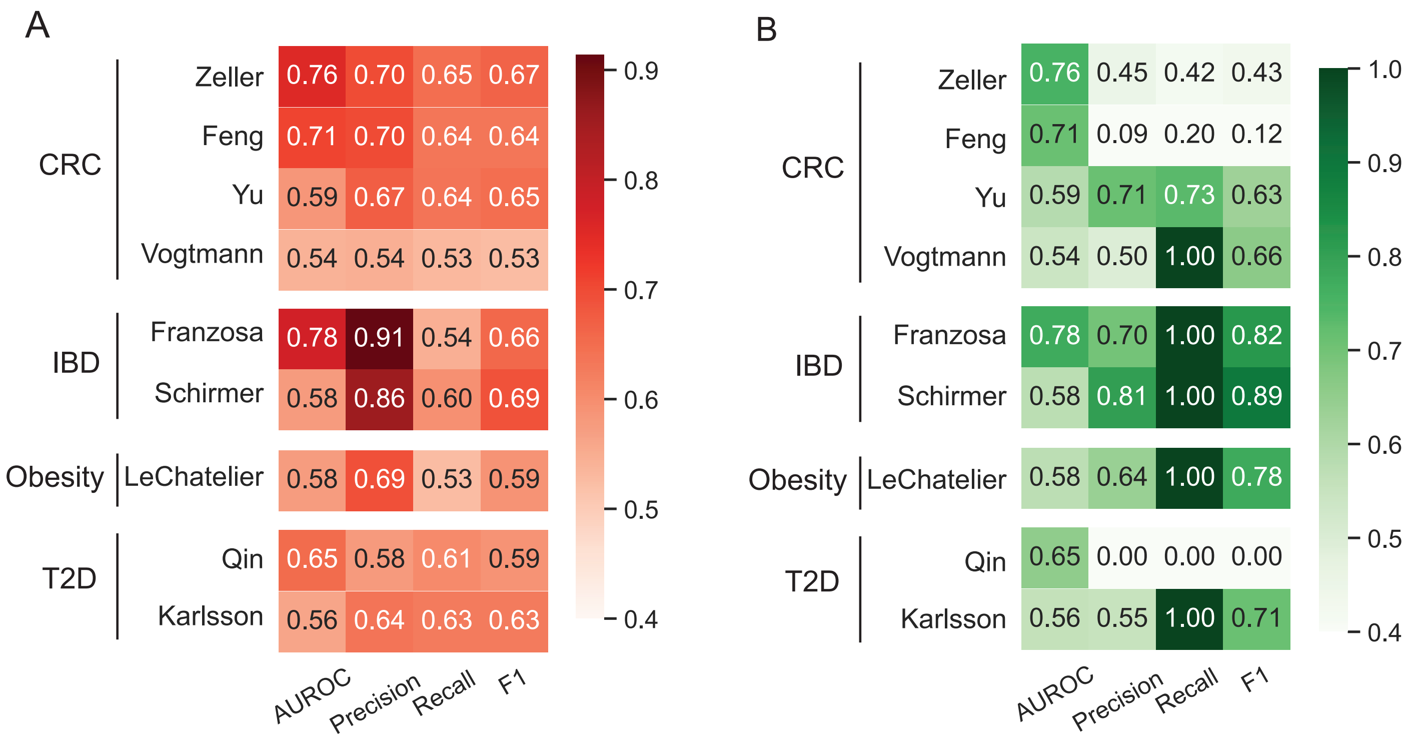

Figure S12. Performance metrics of logistic regression and support vector machine (SVM) models. **(A)** A heatmap of area under the receiver operating characteristic curve (AUROC), precision, recall, and F1-scores for logistic regression on the putative human interactors with the microbiomes of each metagenomic study with feature selection and grid search-based hyper-parameter tuning, evaluated using five-fold cross validation. **(B)** Performance metrics of the SVM classifier.

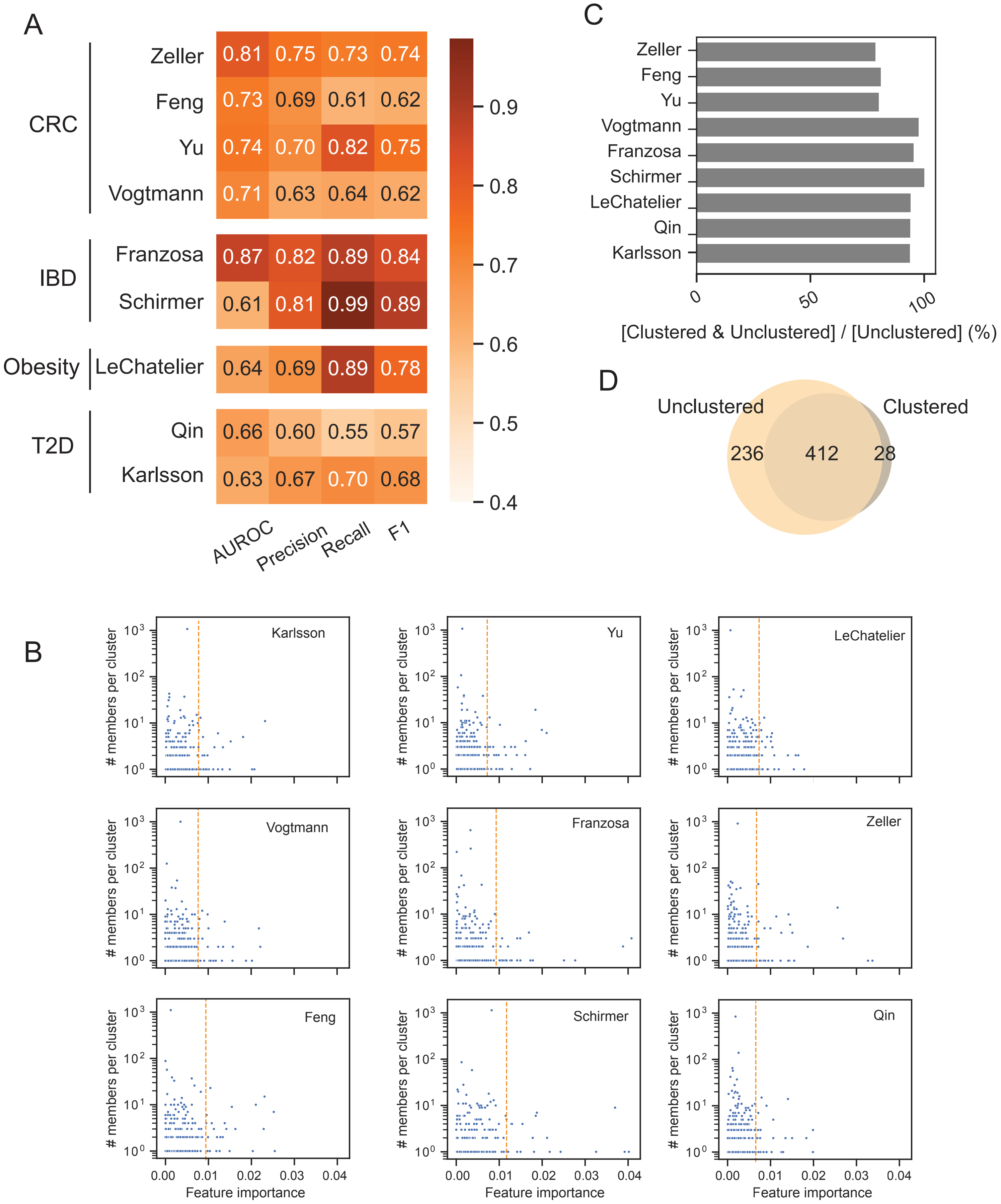

Figure S13. Assessment of the effect of multicollinearity on feature importance rankings.
(A) A heatmap of area under the receiver operating characteristic curve (AUROC), precision, recall, and F1-scores for RF on the putative human interactors with the microbiomes of each metagenomic study with feature selection and grid search-based hyper-parameter tuning, evaluated using five-fold cross validation. (B) The relationship between feature importance and the number of members per cluster. The Orange line is the 90th percentile of feature importances. (C) Percentages of important features in RF trained using clustered human interactors recaptured in RF trained using unclustered data. Features that have Gini importance above the 90th percentile were selected as important features. (D) A Venn diagram of important features identified by RF trained using clustered and unclustered human interactors.

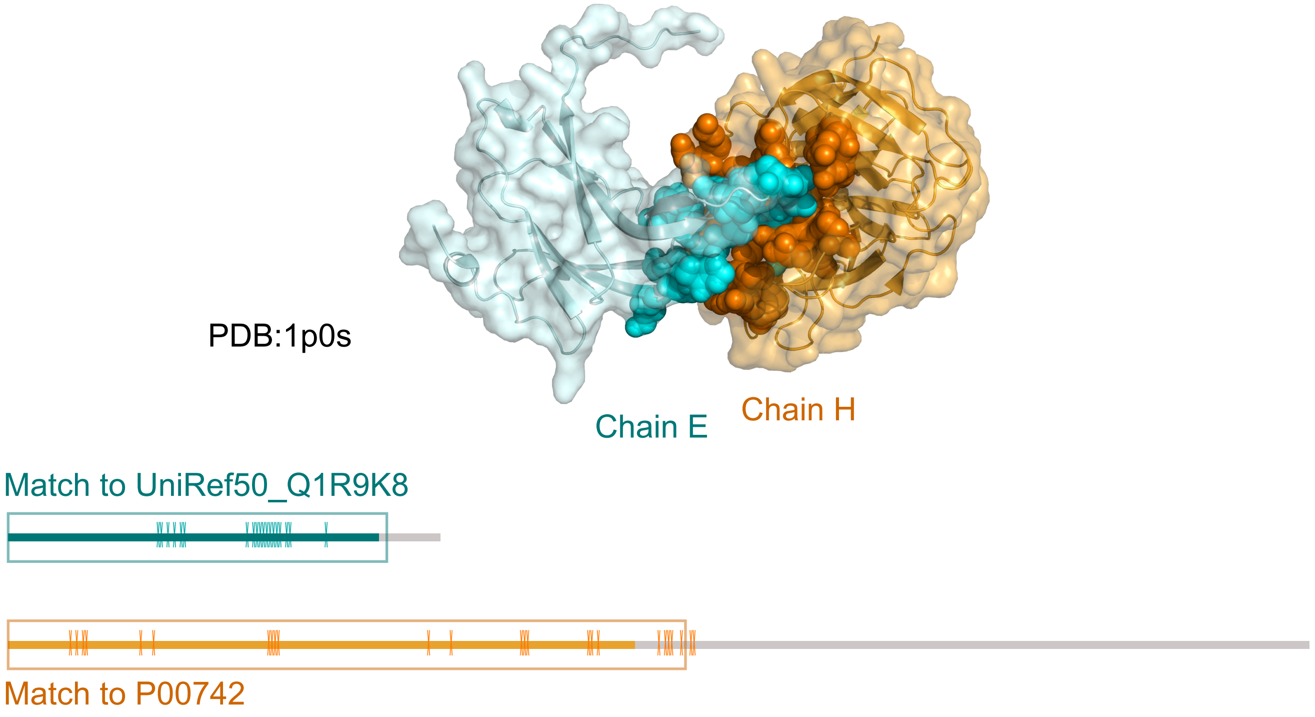

Figure S14. Cocrystal structure of blood coagulation factor Xa in complex with Ecotin M84R. Cluster Uniref50_Q1R9K8 contains several bacterial ecotins detected in human metagenomes. Using BLAST, we found high-quality matches between members of this cluster and the structure 1p0s:E (Ecotin precursor M84R) in the PDB (identity of 97.2%, eval=10^{-75}). Our putative interactor to this cluster, coagulation factor X (P00742) likewise matched structure 1p0s:H (coagulation factor X precursor) (identity of 100%, eval=3.8×10^{-150}). Chain E is shown in blue, and chain H in orange, with their interface residues highlighted as spheres. The linear model of both proteins is shown underneath. The linear model's colored areas indicate the part of the proteins that were crystallized in this PDB, while the greyed-out areas indicate non-crystallized spans. The squares indicate the range of the BLAST match between our query proteins and the PDB reference sequences. Finally, ticks on the linear model indicate the location of interface residues as detected in this model. There are currently not enough published structures to perform this analysis on all interactions involving detected bacterial genes (Fig. S2, Table S6).

Supplementary Tables

Table S1. Extended information on known experimentally verified host-microbiome interactions with evidence for a role in cellular physiology and/or human health.
Information on the interaction detection method for human-microbiome PPIs that have been shown to affect cell physiology and/or human health.

Table S2. Metagenomic samples used in this research.
For each study, we list the sample numbers and labels in the cohort study.

Table S3. Disease-associated human-microbiome PPIs.
Human-microbiome PPIs are listed according to their UniProt and UniRef50 identifiers, human and bacterial protein names.

Table S4. Number of human interactors according to the source of the experimentally-verified interactors.
The number of human interactors, according to the species sourcing the initial experimentally verified interacting protein.

Table S5. Human interactors that are known drug targets.
For each disease-associated human protein, we list the drug interactor (annotated using DrugCentral and DrugBank) and the study in which it was found to be important.

Table S6. Extended information for bacterial proteins targeting known drug targets in Figure 4.
Bacterial clusters depicted in Fig. 4 are listed with their UniRef number and detected taxa, according to HUMANN3.

Table S7. Cocrystal structures representing interactions in our dataset.
All pairs of detected bacterial proteins and human proteins in the nine metagenomic datasets that have BLASTp matches to two different chains within the same PDB cocrystal structure (totaling 8 bacterial protein clusters and 10 human proteins). This list includes structures with at least one chain exclusive to each bacterial and human proteins.

Table S8. Experimental protein-protein interactions that were used for mapping to microbiomes.
